# The impact of clinical pharmacists’ medication reconciliation upon patients’ admission to reduce medication discrepancies in the emergency department: a prospective quasi-interventional study

**DOI:** 10.1186/s12245-023-00568-z

**Published:** 2023-12-15

**Authors:** Heba Othman Shaker, Ahmed Abdel Fattah Sabry, Asmaa Salah, Gilan Mohamed Ragab, Nahla Ahmed Sedik, Zahraa Ali, Doha Magdy, Asmaa Mohamed Alkafafy

**Affiliations:** Alexandria Main University Hospital, Alexandria, Egypt

**Keywords:** Medication reconciliation, Clinical pharmacist, Medication errors, Drug history list, Patient admission

## Abstract

**Background:**

The role of the clinical pharmacist in medication reconciliation is well established. Upon patients’ admission, the reconciliation service mainly focuses on achieving an accurate and full drug history. This will achieve the best treatment plan and reduce medication discrepancies.

Upon the recent implementation of clinical pharmacy services in the emergency department at Alexandria Main University Hospital, medication reconciliation was one of the most important duties that needed to be focused on.

We hypothesized that clinical pharmacists are able to achieve patients’ drug history lists with higher accuracy than emergency physicians.

**Results:**

A total number of 161 patients were included. Age was 58.59 ± (13.78) years, number of comorbidities was 2.39 ± (1.22) and number of home medications was 4.51 ± (2.72). Clinical pharmacists’ fulfillment of patients’ drug history was significantly more accurate than the emergency physicians (75.16% and 50.3% of the total number of revised patients’ profiles respectively). The clinical pharmacists could put a written copy of the accurate patients’ drug history list in only 50.93% of the revised patients’ profiles. Five hundred eighty-five medication discrepancies were detected which represent an average of 3.63 discrepancies/medication sheet. Medications at Transitions and Clinical Handoffs (MATCH) Toolkit for medication reconciliation and the National Coordinating Council for Medication Error Reporting and Prevention (NCC MERP) index were used to categorize discrepancies. Categories A, B, and C represented (66.5%), while categories D, E, and F represented (33.5%) of the total discrepancies. There was a significant direct relationship between the total number of discrepancies and both the number of comorbidities and the number of drugs administered before hospital admission.

**Conclusion:**

The clinical pharmacists are the main members of the emergency health care team. One of their fundamental services is medication reconciliation. The establishment of a complete drug history list and physicians’ discussion about the current treatment plan can obviously detect and reduce medication errors.

**Trial registration:**

NCT04395443. Registered 16 May 2020.

## Introduction

Medication reconciliation is one of the essential and fundamental healthcare services; it is one of the medication management procedures accredited by national and international quality organizations [1–3]. The role of the clinical pharmacist in medication reconciliation on admission, transition of care between different departments, and discharge is well established [3–5].

Medication reconciliation on hospital admission is done first through accurate recording and documentation of a patient’s drug history. Missed or incorrect drug history lists obtained from patients or their family members will lead to inaccurate treatment plans and then different types of medication discrepancies in the hospital medication sheet. Patient history documentation upon admission is the responsibility of all the emergency healthcare team, particularly the clinical pharmacist who has enough time and good skills in patient interviewing [3–5].

The emergency clinical pharmacy is an ongoing and interesting specialty. Until the date of study registration, Alexandria Main University Hospital was the first and only university hospital in Egypt that had an emergency clinical pharmacist’s team. As one of the fundamental tasks, the clinical pharmacy team started a medication reconciliation service.

## Aim

We hypothesized that drug history lists obtained by clinical pharmacists were more accurate than those obtained by emergency physicians.

The primary outcome was the detection of the difference between drug history lists established by the clinical pharmacists and those by emergency physicians. Other outcomes were documenting drug history list copies in the patients’ profiles and finally categorizing the medication errors detected.

## Methods

Approval of the medical ethics committee of Alexandria Faculty of Medicine was granted. The study was registered in clinicaltrials.gov (protocol ID: NCT04395443).

Informed consent was not necessary as medication reconciliation is considered to be an essential task of clinical pharmacists upon patients’ admission.

### Study design

The study was a comparative prospective quasi-one-arm interventional study during the period from 25 September 2020 to 30 November 2020. The study was conducted in the emergency medicine department at Alexandria Main University Hospital.

- Five well-trained emergency clinical pharmacists started the medication reconciliation process on admission. It was done through interviews with the patients or their families during the morning and evening shifts.
  The following items were documented of all patients included in the study*:*
  - Patient characteristics including age, sex, and comorbidities
  - Past medical history and preadmission medications were identified from the patients or their families through interviews, revising previous prescriptions and hospital records.
  - Preadmission medication history included medications’ trade names, doses, frequency, route of administration, and treatment duration, in addition to consumption of vitamins or herbs.
  - Data related to medication discrepancies were measured through a comparison between each patient’s drug history already presented in the profile and the drug history taken by the clinical pharmacists.
- The pharmacists established a hard-written copy of the accurate medication history list for each patient; and discussed it with the treating physician to rearrange the treatment plan.
- Finally, the pharmacists measured the detected medication discrepancies and categorized them using the Medications at Transitions and Clinical Handoffs (MATCH) Toolkit for Medication Reconciliation approved by the agency for health care research and quality and to the National Coordinating Council for Medication Error Reporting and Prevention (NCC MERP).index. It categorizes medication errors into 9 categories: A (no error, capacity to cause error), B (error occurred that did not reach the patient), C (error that reached the patient with no harm), D (error that reached the patient and needed monitoring and or intervention to confirm if no harm), E (error occurred, caused temporary harm and needed intervention), F (error occurred, caused temporary harm and needed prolonged hospitalization), G (error occurred, caused permanent harm), H (error occurred, needs intervention to sustain life), I (error occurred and caused death) [6, 7].

### Inclusion criteria

- Patients with one or more chronic disease/drug.

### Exclusion criteria

Patients who cannot communicate or have no family members.

### Sample size calculation and statistical analysis

#### Sample size justification

In the beginning, a pilot study of 20 patients was carried out and the resulting proportions for the primary outcome in the two arms were 0.5 in case of history taken by physicians (PA) and 0.75 in case of history taken by clinical pharmacists (PB).

When the absolute effect size (δ) is = 0.524, Beta = 0.2, Alpha = 0.05, Kappa = 1.

So the resulting sample size was 58 patients for each arm adding 10% dropouts to reach 64 patients required in each arm to reach 80% power of study.

Power calculation analysis showed that increasing the sample size can increase the power of analysis reaching the maximum sample size of 88 patients for each group, any increase in sample size after that will not alter the power of the study.

So the decision was to include the eligible patients admitted during a period of 2 months, they were 161 patients.

#### Data collection and management

The study was carried out on 161 patients admitted to the emergency department at Alexandria Main University Hospital. Patients’ baseline characteristics including age, sex, and comorbidities had to be documented. After discussing the protocol and the objective of the study and after data collection and verification, all data was fed to statistical analysis using R Software version 3.5.2 (2018-12-20)– “Eggshell Igloo” and the appropriate statistical tests were carried out.

### Analytical statistics

- Chi-square test: to examine the relationship between two categorical variables.
- Regression model: a statistical method used to determine the strength and character of the relationship between one dependent variable and a series of other variables (known as independent variables).

#### Level of significance

The statistical analysis was based on a two-tailed test using a level of significance for analysis at *p* ≤ 0.05. All suitable graphs and tables were done using R Software version 3.5.2 (2018-12-20)– “Eggshell Igloo”.

#### Descriptive statistics

- Descriptive analysis for quantitative data includes mean, standard deviation, and range for normal distributed variables.
- When normal distribution was violated, the median and interquartile range were used instead of the mean and standard deviation.
- For qualitative categorical variables; frequency and percentage were applied.

## Results

Patients’ baseline characteristics are described in Table 1.

**Table 1:** Patients’ baseline characteristics all over the study sample

	Study population (*N* = 161)	
Age	Mean (± SD)	58.59 ± (13.78)
Gender	Male	92 (57%)
	Female	69 (43%)
Number of comorbidities	Mean (± SD)	2.39 ± (1.22)
Number of drugs administered before hospital admission	Mean (± SD)	4.51 ± (2.72)

*SD* standard deviation

The comparison between the accuracy of the patient drug history list taken by physicians and clinical pharmacists was shown in Table 2 as the primary outcome.

**Table 2:** Comparative analysis between the accurate patient drug history list taken by physicians and taken by clinical pharmacists

	Prescriptions with accurate patient drug history list		*P* value
	Yes	No	
Taken by physicians	81 (50.3%)	80 (49.7%)	< 0.001^***^
Taken by pharmacists	121 (75.16%)	40 (24.85%)	

***significant relation

Accurate patient drug history lists taken by the pharmacists were significantly higher than those taken by the physicians (*P* value < 0.001).

Almost half of the accurate patient drug history lists could be documented by the clinical pharmacists in the patient profile as hard-written copies and the rest of the lists could not be documented in the patient profile and verbally informed to the physicians to document them later on. This was represented in Table 3 as the secondary outcome.

**Table 3:** Comparative analysis between documented and verbally informed drug history list

	Study population *N* (%)	*P* value
Documented	82 (50.93%)	0.81
Verbally informed	79 (49.07%)	

*N* number

The clinical pharmacists measured the total number of medication discrepancies as the tertiary outcome and categorized them according to the MATCH Toolkit and NCC MERP index in Table 4.

**Table 4:** The medication discrepancies and their categories using the MATCH Toolkit and NCC MERP index

Discrepancies category	Total number of discrepancies *N* = 585 (%)	*P* value
A	30 (5.128205%)	< 0.0001^***^
B	303 (51.79487%)	
C	56 (9.57265%)	
D	134 (22.90598%)	
E	42 (7.179487%)	
F	20 (3.418803%)	

*N* number

***significant relation

The total number of medication discrepancies was 585 detected all over the 161 medication sheets reviewed. It represents an average of 3.63 discrepancies/medication sheet.

Category B has significantly higher frequency and percentage among those discrepancies than other categories representing about (51.7%) of the total discrepancies followed by category D (22.9%) and then category C (9.5%).

Moreover, there is about 5% of the total discrepancies detected in category A.

Table 5 shows the association between the total number of discrepancies, age, sex, number of comorbidities, and number of drugs administered before hospital admission.

**Table 5:** Linear regression models for the association between numbers of discrepancies with other parameters

	Simple linear regression with total number of discrepancies		Multiple linear regression with total number of discrepancies	
	ß	*P* value	ß	*P* value
Age	0.01151	0.442	0.001756	0.843
Sex (male)	− 0.08333	0.841	0.170801	0.493
**Number of comorbidities**	1.1961	< 0.0001^***^	0.099488	0.460
**Number of drugs administered before hospital admission**	0.77476	< 0.0001^***^	0.746366	< 0.0001^***^

*ß* < regression coefficient

^***^Significant relation

The association between categories of discrepancies, age, sex, number of comorbidities, and number of drugs administered before hospital admission was shown in Table 6.

**Table 6:** Linear regression models for the association between categories of discrepancies with other parameters

Discrepancies category	Simple linear regression models		Multiple linear regression models	
	ß	*P* value	ß	*P* value
Category A				
Age	0.00169	0.700866	− 0.06767	0.812907
Sex (male)	0.15	0.241499	0.000317	0.943842
No. of comorbidities	0.04796	0.353877	0.164553	0.211559
No. of drugs administered before hospital admission	0.020354	0.381741	0.043664	0.538046
Category B				
Age	0.013658	0.25461	− 0.53876	0.421851
Sex (male)	0.274845	0.432308	0.003968	0.706381
No. of comorbidities	0.659373	< 0.001^***^	0.380824	0.21731
No. of drugs administered before hospital admission	0.413704	< 0.001^***^	0.100928	0.543633
Category C				
Age	0.000716	0.898709	0.027131	0.937665
Sex (male)	− 0.22143	0.17585	< 0.001	0.991431
No. of comorbidities	0.138973	0.034548^***^	− 0.21519	0.178353
No. of drugs administered before hospital admission	0.118561	< 0.0001^***^	− 0.08425	03279
Category D				
Age	0.00336	0.671958	− 0.01567	0.974278
Sex (male)	− 0.26491	0.251519	0.000556	0.941957
No. of comorbidities	0.339792	0.000203^***^	− 0.19698	0.378541
No. of drugs administered before hospital admission	0.179837	< 0.0001^***^	0.115272	0.3394
Category E				
Age	− 0.00396	0.232738	0.34822	0.102965
Sex (male)	0.064907	0.502844	− 0.00499	0.136951
No. of comorbidities	0.036096	0.355593	0.089069	0.36268
No. of drugs administered before hospital admission	0.036716	0.035643^***^	− 0.02118	0.6879
Category F				
Age	0.001137	0.638184	0.097558	0.534634
Sex (male)	− 0.03416	0.62795	0.001573	0.524103
No. of comorbidities	− 0.01857	0.513903	− 0.05322	0.461198
No. of drugs administered before hospital admission	0.006109	0.63306	− 0.05605	0.15118

*No* number

*ß* < regression coefficient

^***^Significant relation

## Discussion

- The current prospective quasi-study focuses on the role of the clinical pharmacist in medication reconciliation in the emergency department. To our knowledge and till the date of study registration, it is the first Egyptian study that represents the role of emergency clinical pharmacists generally and includes medication reconciliation service in particular. The study has a novel point of categorizing the detected medication errors using the NCC MERP index. This categorization is the most suitable as it illustrates the occurrence of errors and the degree of harm.

Significant improvement in the accuracy of the patients’ drug history lists taken by the clinical pharmacists compared with those taken by the physicians just upon admission is achieved. One hundred twenty-one out of 161 reviewed profiles (75.16%) compared to 81 out of 161 (50.3%) (*p* value < 0.001).

- The difference might be explained by the busy and critical environment in the emergency department. The physicians focus on rapid examinations, assessment, and patients’ stabilization. It is difficult for emergency physicians to take a complete rapid accurate drug history from the patients or relatives in these acute and life-threatening situations. Clinical pharmacists have more time, more medication evaluation experience, and fewer responsibilities in the emergency department which enables them to do more detailed interviews at the time of admission.
- It is common in many hospitals that it takes 24 h to complete the patients’ drug history list [8]. In this study, medication reconciliation was done just upon patients’ admission which is important for continuing or withholding certain medications during hospitalization.
- It is noticed that 100% accuracy could not be reached either in lists taken by physicians or clinical pharmacists. This is regarded as the limited resources of information in some cases. The pharmacists obtain drug history information from patients /their family members, the bag of medications, and available medical records or reports. If one of them is missing at the time of admission, they consider the drug history list incomplete.
- It is also important to put a hard copy of the accurate drug history lists inside the patients’ profiles as the electronic system is not completely implemented in the hospital. Unfortunately, it is achievable in about 50% of the profiles in the study. As mentioned above about the busy environment of the emergency department, many of the profiles were out of reach. Blood bank, medical registry, and other consultants usually need them to take data or document their notes officially. The clinical pharmacists sometimes could not get the profile again. In about 50% of cases, they informed the physicians verbally with lists they reached while physicians were writing notes to document them themselves later on in the patients’ profiles.

There were in total 585 medication discrepancies detected all over the 161 medication sheets reviewed which represent an average of 3.63 discrepancies/medication sheet.

The results show that errors in categories A, B, and C were 389 out of 585 errors (66.5%), while errors classified as D, E, and F were 196 out of 585 (43.5%).

The number of comorbidities shows a significant relation with the total number of medication discrepancies only in univariate analysis .The number of home drugs (drugs administered before admission) maintain a consistent and significant positive relationship with discrepancies in both uni and multivariate analyses.. This could reflect that patients with a higher complexity of treatment or a greater burden of illness are at more risk. Subgroup categorization of errors did not result in significant relation between each category and either age, sex, number of comorbidities and number of home drugs after multiple linear regression analysis. This paragraph clarifies examples of medication errors. Category A includes some events as high admission rates or many accident admission cases which could increase the capacity to cause medication errors. Examples of category B are inaccurate prescribed doses that were detected before administration. While category C involves a delay in the daily administration time of statin, it did not cause patient harm. When a delay in the daily administration time of an antihypertensive drug was detected, it was classified as D; it needed monitoring to confirm that there was no harm. A missed dose of an antipsychotic in a patient belongs to category E as it caused agitation and needed IV haloperidol administration.

Regarding the errors in category F, two cases will be discussed in detail. In the first patient, there were many differential diagnoses that were suspected by the emergency physicians after the ECG examination. The patient denied taking any medications. Upon his relatives’ detailed interview with the clinical pharmacist and reviewing all drug history. They proved his intake of many digoxin tablets. The physicians confirmed toxicity, started the right treatment plan and the patient improved.

The second was suspected as an opioid overdose case. The patient was admitted with a disturbed level of consciousness and had many IV injection marks. But INR was elevated and CT brain showed hemorrhage. After the clinical pharmacist’s discussion with his family members, they discovered the use of warfarin. So the physicians could explain the lab and radiological findings and start the management of warfarin toxicity.

Of note, there was no patient in this study actually died or suffered from permanent harm. It was achieved through early detection of medication discrepancies, rapid communication between the physicians and the clinical pharmacists, and rapid interventions.

There are some studies that also refer to the medication reconciliation process as one of the clinical pharmacists’ services. Their results align with ours that clinical pharmacists can establish more accurate drug history lists than other healthcare providers. The number of discrepancies detected is a common outcome in all studies but each one classifies them in a different way.

Buckley et al. applied a program in their hospital comparing drug history lists obtained by clinical pharmacists or pharmacy technicians versus other health care teams. They focused mainly on general medical-surgical wards. The researchers did not calculate the percentage of accuracy of the two groups. Their study documented 4699 medication discrepancies in 517 patients. The classification of medication errors was done according to type (wrong dose, wrong route, wrong frequency…). Proximal causes leading to medication error were analyzed. Based on the identification and resolution of medication errors, a sensitivity analysis confirmed that this program is cost-saving [9].

Lombardi NF et al.’s cross-sectional study analyzed the discrepancies identified during medication reconciliation in cardiology units on 24 patients. The discrepancies were classified according to (intentional or unintentional) and to types (omitted drugs, duplicate therapy, changes in doses…) [10].

- Giannini O et al.’s study also includes a comparison between the best possible medication history achieved by the pharmacists and the history written in the profile of 100 patients in the internal medicine ward. Their results included the average time of interviews and classified the sources used to get an accurate medication history list. Medication discrepancies were classified as (omitted drugs, added drugs, incorrect drug strength, formulation…) and clinically relevant or non-relevant. It shows a similar outcome to ours that the number of drugs used before admission was associated with an increased number of discrepancies in a multivariable Poisson regression model [11].

### The strengths of the current study

1. According to the available data, it is the first study done by a specialized clinical pharmacist team in the emergency department in Egypt.
2. A relatively large number of patients are included.
3. It focuses on applying medication reconciliation service just upon patients’ admission. This is crucial for preventing the occurrence of many errors and overcoming the errors leading to permanent harm or death.
4. The study is a quasi and also includes a descriptive analysis.
5. The medication errors detected were classified according to the MATCH Toolkit for medication reconciliation and (NCC MERP**)** index. This index describes the occurrence, significance, and clinical outcome of each error.
6. A Linear regression analysis was done to measure the effect of many important covariates (age, sex, number of comorbidities, and number of drugs administered before hospital admission) on the number of discrepancies in each error category.

## The limitations

1. Further analysis of the effect of potential factors (the physicians and pharmacists’ years of experience, the time spent during the interview) on the primary outcome was not done.
2. Drug history lists hard copies were successfully attached with only 50% of the reviewed patients’ profiles.
3. The drug history information sources are incomplete in some cases.
4. No economic analysis was done to show the cost-saving benefit of applying the reconciliation service.

## Conclusion

The clinical pharmacists can provide many services in the emergency department, especially medication reconciliation.

Medication reconciliation and an accurate drug history list in an emergency are essential in avoiding medication errors and targeting the best patient management plans. Clinical pharmacists have the time and the skills to perform that through rapid communication and thoughtful discussions with physicians.

This service is of great importance to patients with a history of many preadmission drugs who are at greater risk of medication errors.

Implementation of the new national health insurance system in Egypt will provide an electronic profile for each citizen including his past medical history. It will facilitate the medication reconciliation process.

## Data Availability

The datasets used and/or analyzed during the current study are available from the corresponding author on reasonable request.

## Abbreviations

MATCH: Medications at Transitions and Clinical Handoffs
NCC MERP: National Coordinating Council for Medication Error Reporting and Prevention
ECG: Electrocardiography
IV: Intravenous
INR: International normalized ratio
CT: Computerized tomography
